# Which aspects of education are health protective? a life course examination of early education and adulthood cardiometabolic health in the 30-year study of early child care and Youth Development (SECCYD)

**DOI:** 10.1186/s12889-024-18560-4

**Published:** 2024-04-19

**Authors:** Maria E. Bleil, Glenn I. Roisman, Deven T. Hamilton, Sophia W. Magro, Bradley M. Appelhans, Steven E. Gregorich, Cathryn Booth-LaForce, Robert C. Pianta

**Affiliations:** 1 Department of Child, Family, & Population Health Nursing, University of Washington, 98195 Seattle, WA Box 357262, USA; 2 Institute of Child Development, University of Minnesota, Minneapolis, MN USA; 3 Center for Studies in Demography & Ecology, University of Washington, Seattle, WA USA; 4 Department of Family and Preventive Medicine, Rush University Medical Center, Chicago, IL USA; 5 Department of Medicine, University of California San Francisco, San Francisco, CA USA; 6 School of Education & Human Development, University of Virginia, Charlottesville, VA USA

**Keywords:** Socioeconomic status (SES), Education, Income, Cardiometabolic health, Cardiovascular risk factors, Health behaviors, Health disparities, Social skills, Academic achievement, Student-teacher relationship, Classroom emotional quality, Classroom instructional quality, Life course, Study of early child care and Youth Development (SECCYD)

## Abstract

**Background:**

Past research describes robust associations between education and health, yet findings have generally been limited to the examination of education as the number of years of education or educational attainment. Little is known about the specific features or processes underpinning education that are health protective. The objective of the current study was to address this gap by examining specific aspects of early education pertaining to student characteristics and experiences, as well as features of the classroom environment, in predicting cardiometabolic health in adulthood.

**Methods:**

Subjects were 1364 participants in the NICHD Study of Early Child Care and Youth Development (SECCYD, 1991–2009) and recent SECCYD 30-year follow-up, the Study of Health in Early and Adult Life (SHINE, 2018–2022). Models examined individual education indicators (student social skills, student-teacher relationship quality, and classroom emotional and instructional quality in the period of elementary school and student academic performance between ages 54 months and 15 years) in relation to a composite of cardiometabolic risk in adulthood (ages 26–31), reflecting central adiposity, blood pressure, insulin resistance, inflammation, and dyslipidemia. Models were adjusted for key explanatory factors including socio-demographics, infant characteristics, parental socioeconomic status (SES), and child health status. Follow-up analyses were performed to test potential mediators of early education effects on adult health, including adult SES (educational attainment, household income) and health behaviors (diet quality, activity level, sleep duration, smoking).

**Results:**

In adjusted models, results showed greater student social skills, indexed by a mean of annual teacher ratings between kindergarten and 6th grade, predicted lower cardiometabolic risk in adulthood (β=-0.009, *p* <.05). In follow-up analyses, results showed the protective effect of student social skills on cardiometabolic risk may be mediated by adult income (β=-0.0014, *p* <.05) and diet quality (β=-0.0031, *p* <.05). Effects of the other early education indicators were non-significant (*p*s > 0.05).

**Conclusions:**

Findings point to the potential significance of early student social competence as a link to long-term health, possibly via the acquisition of resources needed for the maintenance of health, as well as through engagement in health behaviors supporting healthy eating. However, more research is needed to replicate these findings and to elaborate on the role of early student social competence and the pathways explaining its effects on cardiometabolic health in adulthood.

**Supplementary Information:**

The online version contains supplementary material available at 10.1186/s12889-024-18560-4.

## Background

The socioeconomic status (SES) of individuals has a profound impact on their health, accounting for growing disparities in health in the US and globally [1]. Educational status, a component of SES, shapes employment and income opportunities and is itself associated with myriad health and disease risk indicators [2–6]. Evidence shows the protective effects of education are large in magnitude, persist over the life course, and are mostly invariant across sex and race [7]. Moreover, effects have been observed across countries, time periods, and a variety of health outcomes [7]. To highlight just some of these outcomes, higher educational attainment predicts reduced risk for all-cause and cardiovascular mortality, cardiovascular risk factor profiles, type 2 diabetes, obesity, and inflammation [8–13]. Additional evidence suggests the education-health gradient is increasing [6, 14–16]. For example, trends reveal mortality rates by education are widening overall and within demographic groups, and some of this change is attributable to higher mortality rates in the most disadvantaged groups, especially with respect to new health threats (e.g., opioid overdose) [17]. Taken together, findings indicate there are distinct vulnerabilities among the least educated that must be remediated to lessen growing health gaps, an objective of significant public health concern.

Examination of the behaviors of more highly educated individuals shows they exhibit greater uptake and utilization of new health information [18–20] as was notably demonstrated by higher quit rates in more educated individuals following the 1964 Surgeon General Report on the risks of cigarette smoking [21, 22]. More highly educated individuals are also more likely to engage in health promoting behaviors in general, avoid risky health behaviors, have larger social networks from which to draw support, and put more resources toward health promoting activities, including use of health care in both primary and tertiary care contexts [7, 18, 23–28]. In parallel, inspection of the behaviors of more highly educated parents shows they provide greater monitoring, material support, and household management of routines related to the health habits of their children, as well as model, through their own example, positive health behaviors [29–32]. These efforts transmit intergenerational benefits reflected in the positive health behaviors of their children, including increased physical activity, healthier eating, less sedentary behavior, and reduced health risk behaviors [33–37].

In summary, substantial evidence shows there are prospective links between educational attainment and health as well as distinct characteristics of more highly educated individuals and parents that contribute to better health at the individual level and inter-generationally. Despite this, little is known about the specific features of education that promote health protective behaviors and long-term health outcomes. That is, specific educational experiences or processes, beyond the simple number of years of education, have not been identified in relation to better or worse health. This striking gap in the current literature was recognized by the National Institutes of Health (NIH) in a recent funding initiative (Funding Opportunity Announcement [FOA]: PAR16-080) [38] to which the current study was responsive. This initiative highlighted the need for studies ‘to carefully identify the specific aspects and qualities of education’ that contribute to health as well as the ‘pathways’ and ‘mediating factors’ that explain this potentially causal association. The identification of educational components most salient to health may offer novel opportunities for intervention to specifically target these components, both inside and outside educational settings. Conceivably, this approach may lessen associated disparities in health without requiring attainment of a particular number of years of education per se, an objective, for many, that may not be a realistic path to good health.

When considering specific aspects of the educational milieu that may be candidates for closer inspection in relation to health, the current literature points broadly to individual behaviors of students, relationships between students and their teachers, and features of the classroom environment. Specifically, a large body of work focuses on student social-emotional competence, an individual-level construct describing self-regulatory skills needed for success in school and extracurricular settings [39, 40]. These skills encompass emotional and behavioral self-regulation, self-awareness and control, and interpersonal skills and are all commonly targeted for augmentation in educational environments [41, 42] due to established links with greater academic achievement, improved social relationships, and reduced mental health problems [43–45]. Related work points to the value of understanding relationships between students and their teachers [46–48], showing qualities of positive student-teacher relationships (e.g., higher closeness, lower conflict) predict improved student academic, psychosocial, and attitudinal outcomes with effects persisting over time [49]. Finally, features of classrooms themselves have been examined both with respect to classroom emotional and instructional quality, revealing that higher observed classroom quality predicts a range of adaptive student behaviors and improved academic and social competence outcomes [50–53]. By extension, it is plausible that these same key aspects of early education may facilitate positive health outcomes.

Limitations in the current literature have persisted, in part, because few studies possess data of the appropriate type and resolution to address these gaps. In contrast, the current study, using data from the landmark National Institute of Child Health and Human Development (NICHD) Study of Early Child Care and Youth Development (SECCYD) and recent SECCYD 30-year follow-up study, Study of Health in Early and Adult Life (SHINE), is uniquely positioned to pursue such research questions. The SECCYD (1991–2009) was initiated by NICHD to characterize impacts of early environments, including early educational environments, on domains of child social, emotional, and cognitive development as well as aspects of physical development and health. Subsequently, the follow-up study, SHINE (2018–2022), collected a breadth of social, behavioral, and health status information on the now adult participants [54]. Therefore, longitudinal data are available in multiple areas of early education and adult health and with the use of repeated, multi-method, and gold standard measurement approaches. Moreover, the current study is also uniquely positioned to leverage relevant data to address, at least partially, alternative explanations for observed education-health links. Some of these alternative explanations regard (1) confounding by intergenerational influences such as parental SES; (2) reverse causal mechanisms such as poor child health affecting school performance and future educational opportunities; and (3) ‘third factors’ such as child intelligence affecting both education and health outcomes independently.

Building on the findings and knowledge gaps described above, the current study examined 1364 participants in the NICHD SECCYD and SECCYD 30-year follow-up study, SHINE, to test a series of life course models relating selected early education indicators, including student social skills, student academic performance, student-teacher relationship quality, and classroom emotional and instructional quality, to later health. Here, early education referred to the period of elementary school for the social skills, student-teacher relationship, and classroom quality indicators and the period between ages 54 months and 15 years for the academic performance indicator. The primary goal of the study was to pinpoint which aspects of the early educational milieu are predictive of cardiometabolic health in adulthood, while accounting for potential impacts of confounding, reverse causality, and third factors, by adjusting for socio-demographics, infant characteristics, parental SES, and child health status. For any significant educational predictors, a secondary goal of the study was to identify pathways or mediators of early education effects on adult cardiometabolic health, focusing on adult SES and adult health behaviors. No hypotheses were specified regarding which education indicators may be most important due to a lack of prior research. Accordingly, study findings are expected to add new knowledge about the relative predictive value of specific aspects of early education, a first step in deconstructing the role of education in health promotion.

## Methods

### Participants

Subjects in the current study were participants in the NICHD SECCYD for which there was a recent 30-year follow-up study, rebranded SHINE. The NICHD SECCYD (1991–2009) was a prospective study of children and their families followed between birth and adolescence to examine trajectories of child health and development [55]. The more recent SHINE study (2018–2022) entailed a single in-person study visit conducted between participant ages 26 and 31 years [54] to characterize a breadth of social, behavioral, and health status information in adulthood. See details in Bleil et al. [54] regarding the SHINE study goals, methods, and measures.

NICHD SECCYD recruitment took place in 10 geographically diverse study sites in the United States: Seattle, WA; Madison, WI; Irvine, CA; Pittsburgh, PA; Wellesley, MA; Little Rock, AR; Philadelphia, PA; Morganton, NC; Lawrence, KS; and Charlottesville, VA. In the first 11 months of 1991, all mother-infant dyads of babies born within preselected 24-hour intervals at participating hospitals were screened. NICHD SECCYD exclusions were (1) mother > 1 h from the study site; (2) child being placed for adoption; (3) concurrent participation in another study; and (4) refusal to participate in initial screening. Additional sampling requirements (e.g., 10% single parent households) were imposed to ensure the sociodemographic composition of the final sample (*N* = 1364 families; *n* = 659 girls and *n* = 705 boys) represented the indicated geographies, based on the 1990 US Census. SHINE recruitment retained the original 10 study sites, but augmented recruitment efforts to reach participants who had moved away from the original sites by setting up ancillary sites, paying for participant travel to main or ancillary sites, and creating alternative study protocols that could be completed remotely. SHINE exclusions were (1) lack of written agreement to be re-contacted for future studies (ascertained at the end of the NICHD SECCYD); (2) current pregnancy / breastfeeding (temporary); and (3) current / recent cold or flu symptoms (temporary). On-going rescreening was conducted to monitor changes in temporary exclusions. NICHD SECCYD informed consent and assent were obtained from parents and children, respectively, and the study was approved by the institutional review boards of each university-based study site. SHINE informed consent was obtained from the now adult target participants, and the study was approved by the Human Subjects Division of the University of Washington.

The sample included participants in the NICHD SECCYD (*N* = 1364). However, 5 participants who no longer identified as the sex they were assigned at birth were excluded (i.e., 2 transgender male, 1 transgender female, and 2 non-binary), leaving *n* = 1359 participants in the final analytic sample. Of these participants, 922 were eligible for SHINE because they agreed to be re-contacted for future studies following the conclusion of the NICHD SECCYD, and, of these participants, 700 (75.9%) participated in SHINE.

### Measures

#### Socio-demographics (SECCYD)

Biological sex was coded female vs. male for individuals who continued to identify as the sex they were assigned at birth. Child race/ethnicity was reported by mothers in five categories: Black, Latino, Asian/Pacific Islander (PI), ‘other’ race, and White.

#### Infant characteristics (SECCYD)

Infant characteristics representing stable patterns of emotional/behavioral responding and cognition were indexed by markers of infant temperament and intelligence, respectively. Infant temperament was assessed by mother reports at infant age 6 months using the Revised Infant Temperament Questionnaire [56]. Mothers rated 55 items on a 6-point scale (1 = almost never to 6 = almost always) describing their infant’s activity level, adaptability to changes in the environment, approach to novelty, mood, and intensity. After reversing scoring, the mean of items was taken to form a ‘difficult’ temperament composite score (α = 0.81) with higher values reflecting a more difficult temperament. Infant intelligence was assessed by trained test administrators at infant age 15 months using the Bayley Mental Development Scale [57]. Infants were scored based on their responses to increasingly complex items assessing their sensory-perceptual, memory, learning, classification, generalization, and problem-solving abilities as well as their early verbal skills. A Mental Development Index (MDI) score was calculated for each participant based on their raw score and chronological age with higher values reflecting higher intelligence. In the standardization sample, MDI scores were shown to have high split-half reliability coefficients and to be moderately correlated with subsequent IQ scores [57].

### Parental socioeconomic status (SES) (SECCYD)

Parental SES was indexed by parental education and family income-to-needs ratio. Educational attainment of the mother and father/partner at child age 1 month was assessed by self-report in categories: 1 = less than high school; 2 = high school or general education diploma; 3 = some college or vocational degree; 4 = college degree; 5 = some graduate school or master’s degree; 6 = graduate degree greater than a master’s degree. Family income-to-needs ratio was computed from self-report of family income and family size at child ages 1, 6, 15, 24, 36, and 54 months. Education and income indicators were derived by calculating the mean of mother and father/partner educational attainment and the mean of family income-to-needs ratio across ages, respectively.

### Child health status (SECCYD)

Child health status was indexed by child gestational age, breastfeeding history, BMI percentile (mean), and mother-rated child ‘general’ health (mean). Child gestational age (in weeks) was reported by mothers at child age 1 month. Breastfeeding history was indexed by the total number of months the child was breastfed as determined from mother reports at child ages 1, 6, 15, 24, and 36 months. BMI (weight in kg/height in m^2^) was computed using measurements of height and weight assessed at child ages 24, 36, and 54 months. At each age, BMI percentile was then derived using the 2000 CDC BMI-for-age clinical growth charts (for girls and boys separately), and the mean across ages was calculated. Child general health was rated by mothers at child ages 6, 15, 24, 36, and 54 months in categories (1 = poor to 4 = excellent), and the mean across ages was calculated.

### Early education indicators (SECCYD)

Early education indicators pertained to key student behaviors and experiences as well as aspects of the classroom environment. These indicators included student social skills (teacher rated), student academic achievement (math/reading assessments), student-teacher relationship quality (teacher rated), and classroom emotional and instructional quality (observer rated). ‘Early education’ was defined broadly encompassing all available measures occurring in the period of elementary school for student social skills, student-teacher relationship quality, and classroom emotional and instructional quality, and in the age range, 54 months to 15 years, for student academic achievement. Aggregates of these measures were taken to represent the integrated educational experiences of students over time (see details below).

#### Student social skills

Student social skills were assessed by teacher reports each year between kindergarten and 6th grade using the Social Skills Rating System—Teacher form [58]. The skills in this rating system included behaviors supporting academic and interpersonal success in the classroom setting. Teachers assessed the skills of the target participant in comparison to peers. Teachers rated 30 items on a 3-point scale (0 = never to 2 = very often) in categories of (1) student cooperation, (2) student assertion, and (3) student self-control. ‘Cooperation’ represented rated behaviors in areas of time management, classwork completion and quality, following teacher instructions, organization of school materials, and making transitions between activities. ‘Assertion’ represented rated behaviors in areas of peer engagement, volunteering to help, making friends, offering positive feedback, and joining in activities. ‘Self-control’ represented rated behaviors in areas of controlling anger, willingness to compromise, accepting criticism, handling peer pressure, and being open to peer ideas and classmates from different backgrounds. Standard scores with a theoretical mean of 100 and standard deviation of 15 were calculated to form a student social skills total score for each grade (α = 0.90 to 0.94) with higher scores reflecting greater student social skills. The mean of the 7 grade-level values was then taken to form an overall student social skills score (α = 0.82).

#### Student academic achievement

Student academic achievement was assessed by trained test administrators at child age 54 months, grades 1, 3, and 5, and child age 15 years using subtests from the Woodcock-Johnson Psycho-Educational Battery—Revised [59]. Subtests reflected age-appropriate reading and mathematics content at each assessment. At 54 months, participants completed the Letter-Word Identification and Applied Problems subtests. In grade 1, participants completed the Letter-Word Identification, Word Attack, and Applied Problems subtests. In grade 3, participants completed the Letter-Word Identification, Word Attack, Passage Comprehension, Applied Problems, and Calculation subtests. In grade 5, participants completed the Letter-Word Identification, Passage Comprehension, Applied Problems, and Calculation subtests. Finally, at age 15 years, participants completed the Passage Comprehension and Applied Problems subtests. Standard scores with a theoretical mean of 100 and standard deviation of 15 were calculated for each subtest and the mean was taken to form an academic achievement score for each age/grade level (α = 0.73 to 0.89) with higher scores reflecting higher academic achievement. The mean of the 5 age/grade-level values was then taken to form an overall student academic achievement score (α = 0.92).

#### Student-teacher relationship quality

Student-teacher relationship quality was assessed by teacher reports each year between kindergarten and 6th grade using the Student-Teacher Relationship Scale [60]. Teachers completed 15 items about the degree of conflict and closeness in their relationship with the target participant. After reverse scoring, the mean of items was taken to form a positive relationship total score at each grade level (α = 0.86 to 0.89). The range of scores possible was 15 to 75 with higher scores reflecting higher student-teacher relationship quality. The mean of the 7 grade-level values was then taken to form an overall student-teacher relationship quality score (α = 0.78).

#### Classroom emotional quality and classroom instructional quality

Classroom emotional quality and classroom instructional quality were assessed by trained observers in grades 1, 3, and 5 using the Classroom Observation System [61], which was developed for use in the NICHD SECCYD and later became the Classroom Assessment Scoring System (CLASS) [62]. The classrooms of target participants were observed for 3 h in grade 1 and 6 h each in grades 3 and 5, all in the spring. Eight coding cycles were completed during a typical visit. Observers spent 5 min at the beginning of each coding cycle and 10 min at the end of each coding cycle to observe the classroom environment and take notes. Coders demonstrated adequate reliability compared to a set of ‘gold standard’ codes. Classroom quality ratings from the Classroom Observation System have shown predictive validity (e.g., related to positive student outcomes) and discriminant validity (e.g., unrelated to teacher salary) [63].

To assess classroom emotional quality, trained observers rated the indicated items between 1 (uncharacteristic) and 7 (extremely characteristic) in content areas: (1) classroom positive emotional climate, (2) classroom negative emotional climate, and (3) classroom overcontrol. Classroom positive emotional climate referred to warm and positive aspects of the classroom such as pleasant conversation and positive regard of the teacher toward the students. Classroom negative emotional climate referred to negative aspects of the classroom such as teacher harshness and sarcasm, teacher disapproval and criticism, and negative regard of the teacher toward the students. Classroom overcontrol referred to the extent to which the classroom was rigidly structured with little choice in activities and a high proportion of time spent in teacher-directed activities. After reverse scoring, the mean of items was taken to form a classroom emotional quality total score for each grade (α = 0.57 to 0.83) with higher scores reflecting higher classroom emotional quality. The mean of the 3 grade-level values was then taken to form an overall classroom emotional quality score with a possible range of 1 to 7.

To assess classroom instructional quality, trained observers rated the indicated items between 1 (uncharacteristic) and 7 (extremely characteristic) in content areas that varied by grade. In grade 1, the rated content areas were (1) classroom management (the extent to which the teacher’s expectations were clear and flexible, children understood and followed the rules, and the classroom was operating smoothly and productively); (2) literacy instruction (the extent to which the teacher provided instruction and opportunities related to phonics, reading comprehension, and writing); (3) evaluative feedback (the quality of verbal evaluation of students’ work and ideas); and (4) instructional conversation (the quality of cognitive skills or concepts elicited during teacher-led conversations). In grade 3, the rated content areas were (1) richness of instructional methods (the variety and depth of strategies used by the teacher to present lessons and promote children’s thinking and understanding); (2) classroom chaos (the extent to which the teacher used ineffective control tactics, children were out of control and off-task, and the teacher failed to intervene in situations where an adult was needed); and (3) productive use of instructional time (an indicator of how well the classroom managed time and activities to ensure productivity, student engagement, and instructional efficiency). In grade 5, rated content areas were repeated, including (1) evaluative feedback; (2) richness of instructional methods; (3) classroom chaos; and (4) productive use of instructional time. After reverse scoring, the mean of items was taken to form a classroom instructional quality total score for each grade (α = 0.53 to 0.75) with higher scores reflecting higher classroom instructional quality. The mean of the 3 grade-level values was then taken to form an overall classroom instructional quality score with a possible range of 1 to 7.

### Adult cardiometabolic risk (CMR) composite (SHINE)

The CMR composite included waist circumference (WC), systolic blood pressure (SBP), diastolic blood pressure (DBP), hemoglobin A1c (HbA1c), C-reactive protein (CRP), and high-density lipoprotein (HDL).

#### WC

WC was assessed using a tension-controlled tape measure positioned at the participant’s midpoint between the iliac crest and lowest rib. The measurement was taken on the exhalation and repeated until consecutive measurements were within 0.2 cm. The mean of these final two values was calculated to form the WC indicator.

#### SBP and DBP

SBP and DBP were assessed using a research grade, automated blood pressure monitor, preprogrammed to take three consecutive measurements with one minute in-between readings. The measurement was taken following a 5-minute rest period with the participant seated in a relaxed position and the cuff positioned on the left arm. The mean of these three values was calculated to form separate SBP and DBP indicators.

#### HbA1c, CRP, and HDL

Blood was drawn from the participant’s arm by a trained phlebotomist. Participants were pre-screened for cold and flu symptoms and rescheduled if symptomatic. The blood draw occurred between 7:00 and 10:00 am following an overnight fast and other timed restrictions (e.g., cessation of nicotine). Aliquots were frozen, and assays were performed in batches. HbA1c was assayed using a commercially available ELISA kit (E4656, ABcam, Walthon, MA); the inter-assay coefficient of variation (CV) was 10.4%, and the intra-assay CV was 8.1%. CRP was assayed using a commercially available ELISA kit (KHA0031, Invitrogen/Thermo Fisher Scientific, Waltham, MA); the inter-assay CV was 9.9%, and the intra-assay CV was 6.1%. HDL was assayed using conventional enzymatic methods.

After reverse scoring HDL, all six cardiometabolic risk indicators (WC, SBP, DBP, HbA1c, CRP, and HDL) were standardized, summed, and re-standardized to form the CMR composite.

### Adult pathways (mediators) (SHINE)

Candidate adult pathways between the early education indicators and the adult CMR composite included individual-level SES, indexed by educational attainment and household income, and four health behaviors, indexed by diet quality, activity level, sleep duration, and smoking status.

#### Educational attainment

Educational attainment was assessed by self-report in categories: 1 = no high school diploma; 2 = general equivalency diploma; 3 = high school diploma; 4 = some college but no college degree; 5 = associate’s degree (e.g., AA, AS); 6 = bachelor’s degree (e.g., BA, BS); 7 = some graduate school but no graduate degree; 8 = master’s degree (e.g., MA, MS, MBA); 9 = doctoral degree (e.g., PhD, MD, EdD, DVM, DDS, JD).

#### Household income

Household income was assessed by self-report of total household income divided by the total number of individuals dependent on the income.

#### Diet quality

Diet quality was assessed over the prior 24 h using the computer-based Automated Self-Administered 24-Hour Dietary Assessment (ASA24) [64]. Three interviews were conducted, one in-person and two over the phone, with one interview referencing a weekend day. The interviews were scored using the Healthy Eating Index-2015 (HEI 2015) scoring system developed by the US Department of Agriculture [65]. The overall HEI diet quality scores, based on the US Department of Health & Human Services 2015–2020 Dietary Guidelines, were averaged across interviews to form the diet quality indicator.

#### Activity level and sleep duration

Activity level and sleep duration were assessed using actigraphy. Participants wore a single activity monitor on their right hip during the day for activity assessment and then moved the activity monitor to the wrist of their nondominant hand at night for sleep assessment. The monitor was worn 24 h/day for seven consecutive days. Data were scored using ActiLife software (Actigraph, LLC). Activity level was calculated as total time (in minutes/day) in categories of moderate, vigorous, and very vigorous activity averaged over days to form the activity indicator. Sleep duration was calculated as total sleep time (in hours/night) averaged over nights to form the sleep indicator.

#### Smoking status

Smoking status was assessed by self-report and coded dichotomously in categories of 0 = never smoking vs. 1 = current or past smoking.

### Analytical plan

In separate models, each preselected early education indicator was examined in relation to the adult CMR composite, adjusted for key explanatory factors, including socio-demographics (biological sex, race/ethnicity), infant characteristics (temperament, intelligence), parental SES (parental education, family income-to-needs ratio), and child health status (gestational age, breastfeeding history, BMI percentile, and mother-rated ‘general’ health). The 5 early education indicators were formed by taking means of repeated assessments over the indicated grade levels and included ‘student social skills’ (teacher rated), ‘student academic achievement’ (math/reading assessments), ‘student-teacher relationship quality’ (teacher rated), ‘classroom emotional quality’ (observer rated), and ‘classroom instructional quality’ (observer rated). The adult CMR composite was formed by taking (after reverse scoring HDL) the sum of standardized cardiometabolic risk indicators (WC, SBP, DBP, HbA1c, CRP, and HDL) and re-standardizing. See Measures section for details. Associations were examined using generalized linear models with robust estimators in IBM SPSS Statistics version 28.0.2.0. The reported beta coefficients and the Wald 95% confidence intervals (CI) reflect pooled estimates. For significant education-CMR associations, the relative contributions of the independent predictors to the model goodness-of-fit were assessed using hierarchical partitioning [66].

For each significant education-CMR association, analyses were also performed evaluating potential pathways linking the indicated early education indicator with the adult CMR composite. Examined mediators included adult SES (education, income) and adult health behaviors (diet quality, activity level, sleep duration, and smoking status). All mediation analyses were conducted using the PROCESS v4.2 macro in BMI SPSS Statistics [67] with the exception of analyses involving smoking status. Because the PROCESS macro does not support testing of binary mediators, the examination of smoking status (coded dichotomously) as a mediator was conducted using the R package lavaan 0.6.14 for structural equation modeling [68]. This analysis was run with R 4.3.0.

Multiple imputation was performed for missing data using IBM SPSS Statistics version 28.0.1.0. The NICHD SECCYD had *n* = 1359 observations; 917 observations had no missing values and no variables were missing more than 9.4% of responses. The SHINE follow-up study had *n* = 700 observations; 520 observations had no missing values and no variables were missing more than 24.9% of responses. Observations lost to follow-up were treated as missing data and non-responses were imputed according to established guidelines [69–75]. The imputation used predictive mean matching and two-way interactions in the predictive models and K-match selection equal to 5. The use of predictive mean matching was selected to restrict model predicted values to values observed in the original data. Twenty-eight variables were used in the imputation procedure and 10 imputed data sets were estimated. The mediation analyses, which used data from the SHINE follow-up study for both the dependent variable and the mediators, were restricted to the 520 observations with no missing values due to the correlation of missingness between the dependent variable and the mediators.

## Results

### Descriptives

In Table 1, descriptive statistics are reported for the main variables of interest. Results showed the racial/ethnic composition of the sample was 12.6% Black, 6.1% Latino, 1.4% Asian/PI, 3.5% ‘other’, and 76.4% White. Regarding parental SES, 35.3% and 39.1% of mothers and fathers/partners, respectively, received a college degree or higher and 13.3% of families reported incomes below the federal poverty line. Regarding child health, on average, children were born full term (39 weeks), were breastfed for 4 months, exhibited a healthy weight between ages 24 and 54 months (BMI percentile = 57), and were rated 3.3 (of 4) on a general health rating scale completed by mothers. Examination of these variables in risk categories showed 3.9% were born preterm (< 37 weeks) and 22.0% were born early term (37–38 weeks), 31.9% were not breastfed, 15.7% were overweight or obese at age 24 months, 18.7% at age 36 months, and 24.8% at age 54 months, and 19.3% received at least one ‘poor’ or ‘fair’ general health rating between ages 6 and 24 months. Regarding adult health, on average, values for the individual cardiometabolic risk factors were in healthy ranges. Examination of these variables in risk categories showed 52.9% were above the risk threshold for waist circumference, 29.3% were considered hypertensive, 32.4% were prediabetic or diabetic, 12.0% had CRP values in the risk range, and 27.4% had HDL values in the risk range. Finally, regarding the adult mediators, 55.8% received a college degree or higher and 63.2% reported a household income/dependents <$50,000/year. The average diet quality score (HEI = 50.2) was low compared to an ideal score of 100. The average time engaged in moderate, vigorous, and very vigorous activity was 1.3 h/day, the average duration of sleep was 7.3 h/night, and 27.4% were current or past smokers.

**Table 1:** Sample descriptive statistics

	Mean (SE) or n (%)	95% CI
**Child socio-demographics**		
Biological sex (female)	655 (48.2%)	-
Black	171 (12.6%)	-
Latino	83 (6.1%)	-
Asian/PI	19 (1.4%)	-
‘Other’	47 (3.5%)	-
White	1039 (76.4%)	-
**Infant characteristics**		
Temperament (6 months)	3.18 (0.011)	3.162, 3.205
Intelligence (15 months)	108.29 (0.394)	107.541, 109.041
**Parental SES**		
Mother, college degree+	480 (35.3%)	-
Father, college degree+	487 (39.1%)	-
Families, below poverty (based on mean income-to-needs ratio)	180.2 (13.3%)	-
**Child health status**		
Gestational age (weeks)	39.26 (0.040)	39.184, 39.340
Preterm (< 37 months)	53 (3.9%)	-
Early term (37–38 months)	295 (22.0%)	-
Breastfeeding (months)	4.13 (0.155)	3.826, 4.432
No breastfeeding	433 (31.9%)	-
BMI percentile (mean: 24, 36, 54 months)	57.15 (0.759)	55.755, 58.544
Overweight or obese (24 months)	155 (15.7%)	-
Overweight or obese (36 months)	203 (18.7%)	-
Overweight or obese (54 months)	255 (24.8%)	-
General health rating (mean: 6, 15, 24, 36, 54 months)	3.28 (0.013)	3.254, 3.304
General health rating ‘poor’ or ‘fair’ (6–54 months)	251 (19.3%)	-
**Early educational indicators**		
Student social skills (teacher reported)	102.70 (0.306)	102.035, 103.260
Student academic achievement (math/reading assessment)	106.29 (0.349)	105.635, 106.945
Student-teacher relationship (teacher reported)	63.63 (0.181)	63.295, 63.972
Classroom emotional quality (observer rated)	5.37 (0.019)	5.338, 5.412
Classroom instructional quality (observer rated)	4.20 (0.017)	4.167, 4.235
**Adult cardiometabolic risk indicators**		
WC (cm)	93.21 (0.607)	92.243, 94.167
WC risk (≥ 80 cm women, ≥ 94 cm men)	342 (52.9%)	-
SBP (mm Hg)	116.03 (0.447)	115.341, 116.721
DBP (mm Hg)	73.40 (0.411)	72.828, 73.973
BP risk (SBP ≥ 130 or DBP ≥ 80)	189 (29.3%)	-
HbA1c (%)	5.31 (0.108)	5.183, 5.439
HbA1c risk (≥ 5.7%)	170 (32.4%)	-
CRP (mg/L)	4.58 (0.177)	4.345, 4.814
CRP risk (≥ 10 mg/L)	63 (12.0%)	-
HDL (mg/dL)	53.65 (0.523)	52.945, 54.345
HDL risk (< 50 mg/dL women, < 40 mg/dL men)	144 (27.4%)	-
**Adult pathways (mediators)**:		
Adult educational attainment, college degree+	388 (55.8%)	-
Adult household income/dependents, <$50,000/year	436 (63.2%)	-
Adult diet quality	50.212 (0.429)	49.370, 51.054
Adult activity level	76.157 (2.483)	71.280, 81.035
Adult sleep duration	7.286 (0.042)	7.204, 7.368
Adult smoking status, current/past smoking	190 (27.4%)	-

Means, SEs, and 95% CIs are reported from imputed data for 1359 participants for variables in categories: infant characteristics, parental SES, child health status, early education indicators, and adult cardiometabolic risk indicators. All other values are reported from observed data for NICHD SECCYD participants (*n* = 1359) and SHINE participants (*n* = 700). For observed values, data are missing as follows: 1 missing data for mother education, 113 for father education, 9 for family poverty, 54 for WC, 56 for BP, 176 for HbA1c, 175 for CRP, 175 for HDL, 5 for adult educational attainment, 10 for adult household income, 39 for adult diet quality, 123 for adult activity level, 121 for adult sleep duration, and 6 for adult smoking status. Abbreviations are as follows: *SE* standard error; *CI* confidence interval; *PI* Pacific Islander; *SES* socioeconomic status; *BMI* body mass index; *WC* waist circumference; *SBP* systolic blood pressure; *DBP* diastolic blood pressure; *BP* blood pressure; *HbA1c* hemoglobin A1c; *CRP* C-reactive protein; *HDL* high-density lipoprotein

### Unadjusted analyses

In Table 2, in separate, unadjusted linear regression models, examination of the early education indicators in relation to the adult CMR composite showed greater student social skills (β=-0.015; 95% CI: -0.022, -0.007; *p* <.001), higher student academic achievement (β=-0.012; 95% CI: -0.018, -0.006; *p* <.001), higher student-teacher relationship quality (β=-0.021; 95% CI: -0.037, -0.005; *p* <.05), and higher classroom instructional quality (β=-0.111; 95% CI: -0.216, -0.005; *p* <.05) each predicted lower cardiometabolic risk in adulthood. Higher classroom emotional quality predicted lower cardiometabolic risk in adulthood at the level of a statistical trend (β=-0.110; 95% CI: -0.237, 0.017; *p* =.088).

**Table 2:** In separate models, unadjusted effects of the early educational indicators on the adult CMR composite

	DV: Adult CMR Composite		
	Beta	95% CI	*p*-value
**Predictors**			
**Early educational indicators**			
Student social skills (teacher reported)	-0.015***	-0.022, -0.007	< 0.001
Student academic achievement (math/reading)	-0.012***	-0.018, -0.006	< 0.001
Student-teacher relationship (teacher reported)	-0.021*	-0.037, -0.005	0.012
Classroom emotional quality (observer rated)	-0.110	-0.237, 0.017	0.088
Classroom instructional quality (observer rated)	-0.111*	-0.216, -0.005	0.040

*<0.05, **<0.01, ***<0.001

*CMR* cardiometabolic risk; *CI* confidence interval

### Adjusted analyses

In Table 3, in a parallel set of linear regression models adjusted for key explanatory factors (socio-demographics, infant characteristics, parental SES, and child health status), the pattern of results changed. That is, with adjustment, only student social skills persisted in predicting lower cardiometabolic risk in adulthood (β=-0.009; 95% CI: -0.016, -0.001; *p* <.05). Among the explanatory factors, being female (vs. male) (β=-0.487; 95% CI: -0.639, -0.335; *p* <.001), having parents who are more educated (β=-0.092; 95% CI: -0.175, -0.008; *p* <.05), and having a higher family income-to-needs ratio in childhood (β=-0.038; 95% CI: -0.062, -0.003; *p* <.05) were independent predictors of *lower* cardiometabolic risk in adulthood, while having a higher BMI percentile in childhood (β = 0.006; 95% CI: 0.003, 0.009; *p* <.001) was an independent predictor of *higher* cardiometabolic risk in adulthood.

**Table 3:** Direct effects of the early educational indicator, student social skills, on the adult CMR composite, adjusted for socio-demographics, infant characteristics, parental SES, and child health status

	DV: Adult CMR Composite		
	Beta	95% CI	*p*-value
**Predictors**			
**Socio-demographics**			
Biological sex (female vs. male)	-0.487***	-0.639, -0.335	< 0.001
Black (vs. white)	-0.015	-0.297, 0.268	0.916
Latino (vs. white)	0.101	-0.437, 0.237	0.546
Asian/PI (vs. white)	-0.035	-0.360, 0.291	0.834
‘Other’ (vs. white)	-0.073	-0.454, 0.307	0.700
**Infant characteristics**			
Temperament	0.033	-0.124, 0.190	0.680
Intelligence	0.001	-0.004, 0.006	0.697
**Parental SES**			
Parental education	-0.092*	-0.175, -0.008	0.032
Family income-to-needs ratio	-0.038*	-0.062, -0.003	0.030
**Child health status**			
Gestational age	-0.033	-0.092, 0.026	0.264
Breastfeeding history	0.005	-0.007, 0.017	0.407
BMI percentile	0.006***	0.003, 0.009	< 0.001
General health rating	-0.024	-0.159, 0.110	0.728
**Early education indicator**			
Student social skills	-0.009*	-0.16, -0.001	0.027

*<0.05, **<0.01, ***<0.001

*CMR* cardiometabolic risk; *SES* socioeconomic status; *CI* confidence interval; *PI* Pacific Islander; *BMI* body mass index

**Fig. 1:**
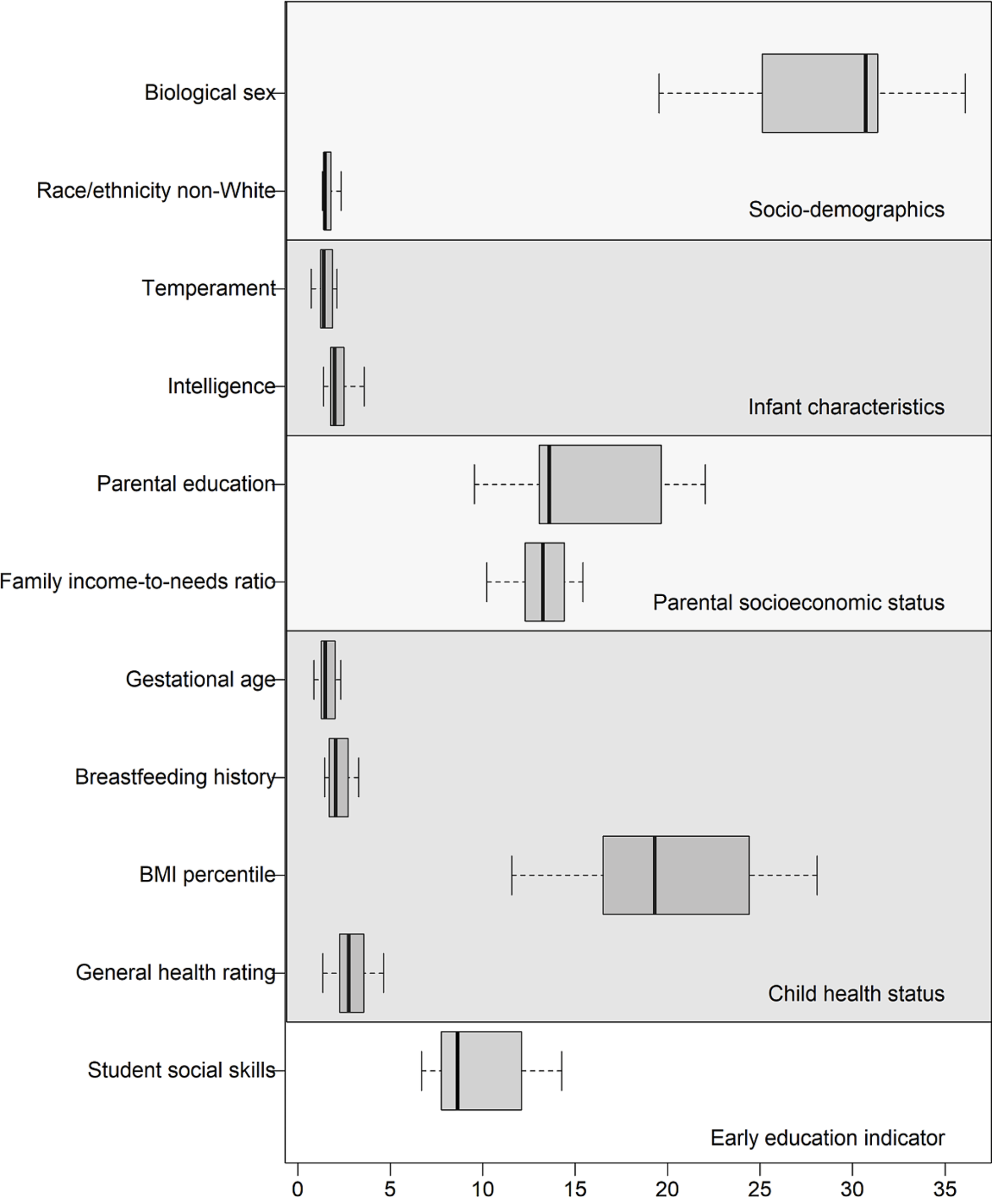
Results are reported from analyses using hierarchical partitioning to determine the relative contributions of the student social skills indicator and the key explanatory factors

In analyses examining the relative contributions of student social skills and the key explanatory factors, a hierarchical partitioning algorithm was used to partition the independent and joint contributions of each predictor to the goodness-of-fit (*R*-squared) of the regression model. In Fig. 1, the proportion of fit that can be independently attributable to each predictor is displayed. Specifically, each boxplot indicates the range of the attributable fraction over the 10 imputed data sets. Reported at the mean of these ranges, student social skills accounted for 9.8% of the independently attributable variance in the adult CMR composite. This compares to the key explanatory factors in which socio-demographics accounted for 30.6%, infant characteristics accounted for 3.8%, parental SES accounted for 28.7%, and child health status accounted for 27.1% of the independently attributable variance in the adult CMR composite.

With respect to the other early education indicators (student academic achievement, student-teacher relationship quality, classroom emotional quality, classroom instructional quality) all associations with the adult CMR composite were non-significant (*p*s > 0.05) (Supplemental Tables 1–4). Among the key explanatory factors in these other models, the pattern of results was similar, highlighting that biological sex, parental SES, and child BMI percentile were independent predictors of cardiometabolic risk in adulthood.

### Mediation analyses

**Fig. 2:**
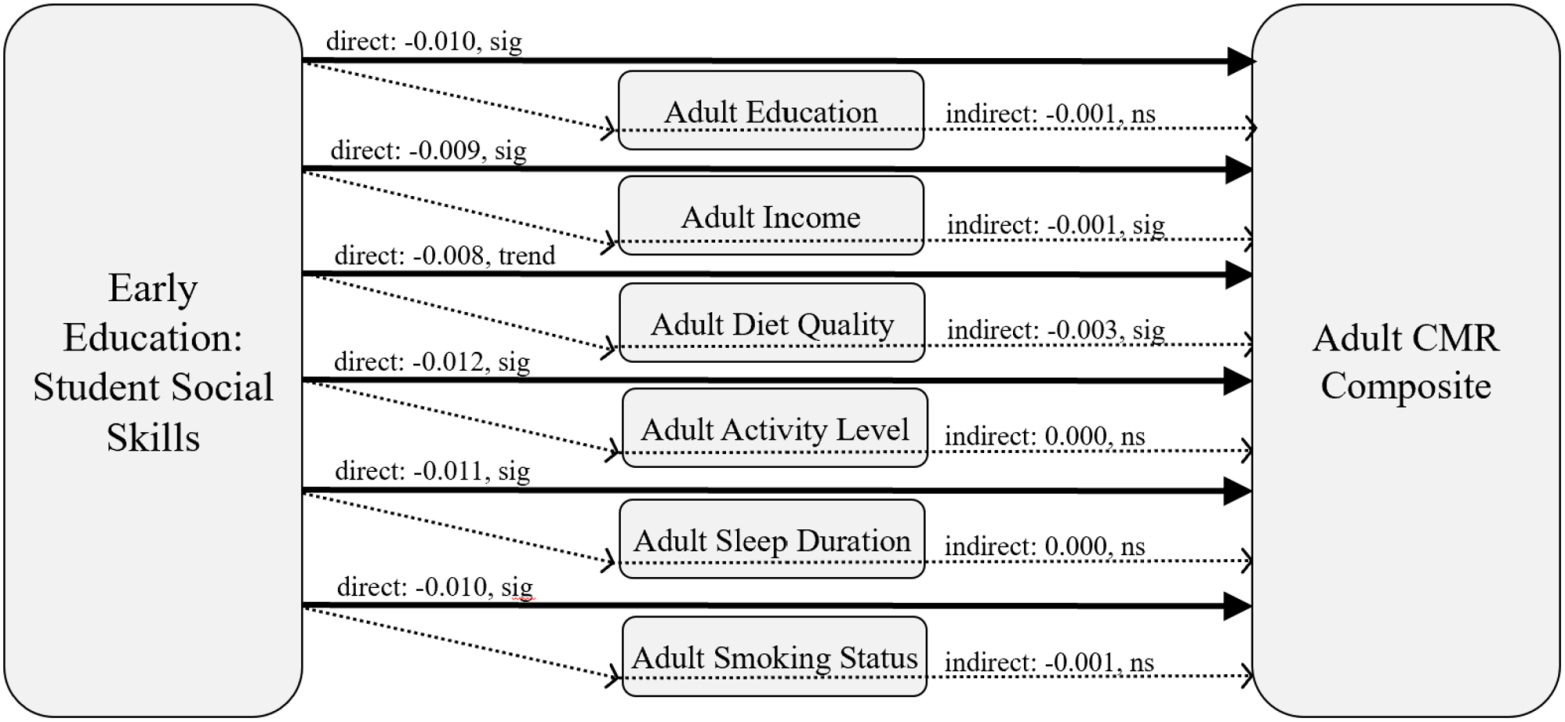
Results are reported from separate regression models adjusted for socio-demographics, family income-to-needs ratio, and child BMI percentile. The solid arrows represent direct effects between the indicated early educational indicator (student social skills) and the adult CMR composite. The dotted arrows represent the indirect (mediated) effects of this indicator on the adult CMR composite via adult SES (education, income) and adult health behaviors (diet quality, activity level, sleep duration, and smoking status). Results suggest protective effects of student social skills on reduced risk for adult cardiometabolic risk are partially attributable to adult SES (greater income) and adult health behaviors (better diet quality)

In Table 4; Fig. 2, to better understand the observed significant association between student social skills and the adult CMR composite, mediation analyses were performed examining potential mechanistic pathways linking these developmental periods. Examined mediators showed significant indirect effects of student social skills on cardiometabolic risk in adulthood via adult income (β=-0.0014; 95% CI: -0.0028, -0.0004; *p* <.05) and adult diet quality (β=-0.0031, 95% CI: -0.0052, -0.0013; *p* <.05). These effects were independent of adjustment for biological sex, race/ethnicity, family income-to-needs ratio, and child BMI percentile. That is, results suggest that the protective effects of student social skills on reduced risk for poor cardiometabolic health in adulthood are partially transmitted by adult SES (greater income) and adult health behaviors (better diet quality), even beyond risks conferred by other early factors (e.g., childhood obesity).

**Table 4:** Mediated effects of the early educational indicator, student social skills, on the adult CMR composite, via adult SES indicators and adult health behaviors, adjusted for socio-demographics, family income-to-needs ratio, and child BMI percentile

	DV: Adult CMR Composite			
Predictors	Effects	Beta	95% CI	*p*-value
Adult education	Direct effect:	-0.0097	-0.0585, 0.0392	0.697
Student social skills mediated by Adult education	Total effect:	-0.0108**	-0.0185, -0.0031	0.006
	Direct effect:	-0.0103*	-0.0184, -0.0022	0.013
	Indirect effect:	-0.0005	-0.0032, 0.0021	ns
Adult income	Direct effect	-3e-6*	-5e-6, -6e-7	0.014
Student social skills mediated by Adult income	Total effect:	-0.0108**	-0.0185, -0.0031	0.006
	Direct effect:	-0.0094*	-0.0171, -0.0016	0.018
	Indirect effect:	-0.0014*	-0.0028, -0.0004	sig
Adult diet quality	Direct effect:	-0.0157***	-0.0225, -0.0089	< 0.001
Student social skills mediated by Adult diet quality	Total effect:	-0.0106**	-0.0183, -0.0030	0.007
	Direct effect:	-0.0076	-0.0152, 0.0001	0.053
	Indirect effect:	-0.0031*	-0.0052, -0.0013	sig
Adult activity level	Direct effect:	-0.0002	-0.0016, 0.0011	0.725
Student social skills mediated by Adult activity level	Total effect:	-0.0121**	-0.0203, -0.0039	0.004
	Direct effect:	-0.0122**	-0.0204, -0.0040	0.004
	Indirect effect:	0.0001	-0.0006, 0.0009	ns
Adult sleep duration	Direct effect:	-0.0570	-0.1370, 0.0229	0.162
Student social skills mediated by Adult sleep duration	Total effect:	-0.0106*	-0.0189, -0.0024	0.012
	Direct effect:	-0.0106*	-0.0189, -0.0023	0.012
	Indirect effect:	0.0000	-0.0007, 0.0007	ns
Adult smoking status	Direct effect:	0.0202	-0.0680, 0.1084	0.654
Student social skills mediated by Adult smoking status	Total effect:	-0.0109**	-0.0185, -0.0032	0.005
	Direct effect:	-0.0103*	-0.0183, -0.0024	0.011
	Indirect effect:	-0.0005	-0.0029, 0.0018	0.656

*<0.05, **<0.01, ***<0.001

sig: The indirect effects are estimated by bootstrap and a p-value is not calculated; inspection of the confidence interval shows it does not include 0, indicating a significant association at the *p* <.05 level or greater. ns: The indirect effects are estimated by bootstrap and a p-value is not calculated; inspection of the confidence interval shows it does include 0, indicating a non-significant association

## Discussion

The objective of the current study was to leverage data from the NICHD SECCYD and SECCYD 30-year follow-up study, SHINE, to examine specific aspects of early education in relation to adult cardiometabolic health. In the primary analyses, findings showed that, of the five early education indicators examined, four were significantly associated with the adult CMR composite in unadjusted analyses, including student social skills, student academic achievement, student-teacher relationship quality, and classroom instructional quality. However, after adjustment for key explanatory factors, only student social skills persisted in predicting the adult CMR composite. That is, greater student social skills, reflecting an aggregate of annual ratings provided by different teachers over an extended period of 7 years (kindergarten to 6th grade), predicted lower cardiometabolic risk in adulthood. In addition, in secondary analyses, findings showed that the protective effect of student social skills on adult cardiometabolic risk was partially mediated by adult income and adult diet quality. The current study adds new knowledge about the potential salience of student social competence and its pathways leading to better adult health. Implications are that future work should continue efforts to pinpoint the health promoting components of education and that potential targeting of such factors may offer new opportunities for early health promotion, even outside of educational settings.

The attenuation of significant associations between student academic achievement, student-teacher relationship quality, and classroom instructional quality and the adult CMR composite in adjusted models indicates these effects were attributable to other key explanatory factors, including socio-demographics, infant characteristics, parental SES, and child health status. It is also noteworthy that, in all five of the final adjusted models, biological sex, parental SES, and child BMI percentile were significant independent predictors of the adult CMR composite. This highlights the strong protective impacts of female biological sex, higher parental SES, and lower child BMI percentile on adult cardiometabolic health and the need to consider these factors when examining the shared and unique effects of early education. Finally, to further characterize these explanatory factors in the context of the student social skills indicator specifically, the relative significance of these factors was examined (see Fig. 1). Interestingly, student social skills accounted for a sizable proportion (9.8%) of the independently attributable variance in the adult CMR composite, exceeding the variance explained by infant characteristics (3.8%) but smaller in size than the variance explained by socio-demographics (30.6%), parental SES (28.7%), and child health status (27.1%).

The emergence of student social skills is consistent with prior studies showing the unique predictive value of social-emotional competence in relation to later outcomes [39, 40]. Such self-regulatory skills have been shown to predict superior academic, social, and mental health outcomes and are commonly targeted in school-based programs [41–45]. In Jones et al. [76], teacher-rated social competence in kindergarten predicted late adolescent and adult outcomes including educational achievement and employment stability as well as reductions in use of public assistance, criminal activity, and substance abuse. The current study extends this work to physical health outcomes, highlighting the importance of understanding the role social competence in a school context appears to play in the long-term maintenance of physical health. As was discussed in the NIH funding initiative [38], to which the current study was responsive, children’s entrance into formal education may establish a foundation for their integration into society more broadly. That is, through school-based socialization efforts and the development of social competencies including social skills, social communications, and social interactions, children may learn how to navigate structured, complex, and hierarchical settings such as the school environment, likely promoting success in future educational and employment contexts. Although speculative, these early experiences may be critical to health for several reasons. Greater social competence may enhance engagement in school, resulting in more years of school attendance and contributing to the known association between educational attainment and health [8–13]. Greater social competence may enhance social networks, a possible source of support for improved health, including through increased access to resources [25, 77–79]. Greater social competence may also support the adoption of social and behavioral norms in a classroom setting, possibly reinforcing a positive self-concept that promotes the uptake of positive behaviors in other health related contexts.

Another possibility is that greater social competence in school may reflect a set of skills that overlaps or builds on capacities needed to enact and maintain positive health behaviors. The student social skills indicator examined in the current study represented an assortment of behaviors such as time management, following through on tasks, transitioning between activities, engaging in social interactions, and exhibiting self-regulation in classroom and social activities. Beyond social competence, these behaviors may be tapping into children’s executive function, defined as a set of cognitive processes that support goal directed behavior, including inhibitory control, working memory, and cognitive flexibility, as well as second order processes such as planning and problem-solving [80]. Prior evidence in adults suggests executive function is an essential feature of healthy lifestyles [81–83] determining health and disease risk over time [84, 85]. Individuals with deficits in executive function show poor adherence to healthy eating and physical activity as well as greater engagement in health risk behaviors [86, 87]. Similar associations between executive function and health behaviors have been observed in children including with respect to consumption of sugar-sweetened beverages, snack food intake, screen time related sedentary behavior, and cardiovascular fitness, with some evidence suggesting these associations may be bi-directional [88–92].

Follow-up mediational analyses in the current study showed effects of student social skills in elementary school may impact adult cardiometabolic health through adult income and diet quality. Adult educational attainment, activity level, sleep duration, and smoking were not statistically significant mediators. This suggests that student social competence may partially operate through the acquisition of resources helpful in the maintenance of health, as well as through engagement in health behaviors supporting healthy eating. This also suggests that educational attainment, per se, was not a central mechanism linking early student social skills and subsequent health outcomes in this sample. These findings are intriguing and point to areas for future investigation to begin to understand how the early educational experiences of children may embed such pathways.

Additional areas for future investigation stem from the literatures mentioned above. These include consideration of the social networks of children of varying levels of social competence as well as the ways friend networks, known to influence health behaviors [93–95], may be operating in these children uniquely. Understanding these structures and processes with respect to social competence, may be particularly informative in identifying ways to harness the power of peer influences and social norms [96, 97] for the advancement of positive health behaviors. In addition, future investigation should consider the intersection of social competence with executive function. Whether there is overlap or additive benefits of being high in both areas would inform potential efforts to augment these skill sets. More broadly, future investigations should also consider how potential intervention targets could be approached outside of educational settings and begin to move beyond the limited focus on educational attainment, a strategy that might reach more children. Finally, although findings from the current study were non-significant for the student academic performance, student-teacher relationship quality, and classroom quality indicators, these constructs should be retained in future investigations. It remains possible that alternative measurement and analytical strategies are needed to better characterize these constructs and their potential relation to adult health outcomes.

A primary strength of the current study was the use of data from the landmark NICHD SECCYD and recent SECCYD follow-up study, SHINE. This longitudinal study of more than 30 years offered the unique opportunity to test a series of life course models using real-time, state-of-the-art methods and measures. The availability of high-resolution longitudinal data focusing on areas of early education is relatively unique, especially regarding the opportunity to examine these variables in relation to similarly high-quality assessments of adult cardiometabolic health and health behaviors. The adult CMR composite represented key components of adulthood cardiometabolic health measured according to best practices in areas of central adiposity, blood pressure, insulin resistance, inflammation, and dyslipidemia. Finally, the current study also possessed data necessary to control for key explanatory factors, potentially accounting for education-health links, including parental SES, child health status prior to school entry, and child factors related to temperament and intelligence. A primary weakness of the current study was the lack of racial and ethnic diversity in the sample limiting the opportunity to examine subgroups of participants and limiting the generalizability of the study findings to non-white children. An additional weakness was the broad analytical approach, which aggregated measures of early education over the period of elementary school without consideration for how the predictive value of these indicators may vary by age or developmental stage—an area requiring future inquiry. Finally, study findings are considered preliminary and require replication with respect to the value of focusing on student social skills specifically as well as the mechanisms of its potential impact on long-term health.

## Conclusions

In summary, growing SES-related disparities in health in the US and globally underpin the public health imperative to identify novel approaches to lessen such gaps. A limitation in the current literature regards the lack of information about how specific aspects of education may contribute to long-term health and disease prevention. The current study examined multiple early education indicators, finding teacher-rated student social skills in elementary school was a main predictor of cardiometabolic health in adulthood, with evidence suggesting adult income and diet quality may play a mechanistic role in transmitting these effects. These findings point to the potential significance of early school-based socialization processes as a link to long-term health, possibly through the acquisition of resources needed for the maintenance of health, as well as through engagement in health behaviors supporting healthy eating. However, more research is needed to replicate these findings and to elaborate on the role of early social competence and the pathways explaining its effects on adult cardiometabolic health. More work is also needed to continue efforts to identify the specific components of education that are health protective and could be targeted for early intervention, even outside of traditional education settings.

### Electronic supplementary material

Below is the link to the electronic supplementary material.

Supplementary Material 1

## Data Availability

Data from the original NICHD SECCYD analyzed in the current study are available online: ICPSR NICHD Study of Early Child Care and Youth Development (SECCYD) Series https://www.icpsr.umich.edu//web/ICPSR/series/233. Data from the SHINE follow-up study analyzed in the current study are not available online due to privacy and ethical restrictions. Researchers interested in working with the team of investigators who led the SHINE follow-up data collection are invited to contact MEB and GIR. Collaborative efforts will be considered under specific conditions, including the scope of work and assurances related to data security and integrity.
