# Supplementary material for: Which aspects of education are health protective? a life course examination of early education and adulthood cardiometabolic health in the 30-year study of early child care and Youth Development (SECCYD)
Source: BMC Public Health. 2024 Apr 19;24:1092. doi: 10.1186/s12889-024-18560-4 (PMC11031877; doi:10.1186/s12889-024-18560-4)
Supplement: Supplementary file 1 — Supplementary Material 1 [file 12889_2024_18560_MOESM1_ESM.docx]

Supplemental Table 1. Direct effects of the early educational indicator, student academic achievement, adjusted for socio-demographics, infant characteristics, parental SES, and child health status.

|  | **DV: CMR Composite** | | |
| --- | --- | --- | --- |
|  | **Beta** | **95% CI** | **p-value** |
| **PREDICTORS:** |  |  |  |
| **Socio-demographics:** |  |  |  |
| Biological sex (female vs. male) | -0.512*** | -0.669, -0.355 | <0.001 |
| Black (vs. white) | -0.030 | -0.334, 0.274 | 0.839 |
| Latino (vs. white) | -0.005 | -0.361, 0.352 | 0.979 |
| Asian/PI (vs. white) | -0.022 | -0.377, 0.333 | 0.901 |
| ‘Other’ (vs. white) | -0.004 | -0.377, 0.369 | 0.982 |
| **Infant characteristics:** |  |  |  |
| Temperament | 0.024 | -0.203, 0.251 | 0.827 |
| Intelligence | 0.001 | -0.004, 0.006 | 0.627 |
| **Parental SES:** |  |  |  |
| Parental education | -0.073 | -0.167, 0.021 | 0.121 |
| Family income-to-needs ratio | -0.037* | -0.072, -0.003 | 0.033 |
| **Child health status:** |  |  |  |
| Gestational age | -0.028 | -0.084, 0.028 | 0.312 |
| Breastfeeding history | 0.003 | -0.010, 0.016 | 0.658 |
| BMI percentile | 0.006*** | 0.004, 0.008 | <0.001 |
| General health rating | -0.028 | -0.193, 0.138 | 0.736 |
| **Early education indicator:** |  |  |  |
| Student academic achievement | -0.005 | -0.012 - 0.002 | 0.172 |

*<.05, **<.01, ***<.001

SES=socioeconomic status; CMR=cardiometabolic risk; CI=confidence interval; PI=Pacific Islander; BMI=body mass index

Supplemental Table 2. Direct effects of the early educational indicator, student-teacher relationship, adjusted for socio-demographics, infant characteristics, parental SES, and child health status.

|  | **DV: CMR Composite** | | |
| --- | --- | --- | --- |
|  | **Beta** | **95% CI** | **p-value** |
| **PREDICTORS:** |  |  |  |
| **Socio-demographics:** |  |  |  |
| Biological sex (female vs. male) | -0.508*** | -0.662, -0.354 | <0.001 |
| Black (vs. white) | -0.002 | -0.319, 0.316 | 0.992 |
| Latino (vs. white) | 0.000 | -0.360, 0.360 | 0.998 |
| Asian/PI (vs. white) | -0.043 | -0.394, 0.309 | 0.811 |
| ‘Other’ (vs. white) | 0.001 | -0.371, 0.372 | 0.998 |
| **Infant characteristics:** |  |  |  |
| Temperament | 0.026 | -0.201, 0.254 | 0.813 |
| Intelligence | 0.000 | -0.004, 0.005 | 0.879 |
| **Parental SES:** |  |  |  |
| Parental education | -0.089* | -0.177, -0.001 | 0.048 |
| Family income-to-needs ratio | -0.039* | -0.073, -0.004 | 0.028 |
| **Child health status:** |  |  |  |
| Gestational age | -0.028 | -0.084, 0.028 | 0.311 |
| Breastfeeding history | 0.003 | 0.011, 0.016 | 0.699 |
| BMI percentile | 0.006*** | 0.003, 0.008 | <0.001 |
| General health rating | -0.039 | -0.207, 0.130 | 0.644 |
| **Early education indicator:** |  |  |  |
| Student-teacher relationship | -0.001 | -0.015, 0.012 | 0.830 |

*<.05, **<.01, ***<.001

SES=socioeconomic status; CMR=cardiometabolic risk; CI=confidence interval; PI=Pacific Islander; BMI=body mass index

Supplemental Table 3. Direct effects of the early educational indicator, classroom emotional quality, adjusted for socio-demographics, infant characteristics, parental SES, and child health status.

|  | **DV: CMR Composite** | | |
| --- | --- | --- | --- |
|  | **Beta** | **95% CI** | **p-value** |
| **PREDICTORS:** |  |  |  |
| **Socio-demographics:** |  |  |  |
| Biological sex (female vs. male) | -0.512*** | -0.671, -0.354 | <0.001 |
| Black (vs. white) | -0.002 | -0.333, 0.329 | 0.990 |
| Latino (vs. white) | 0.003 | -0.354, 0.361 | 0.985 |
| Asian/PI (vs. white) | -0.039 | -0.396, 0.318 | 0.828 |
| ‘Other’ (vs. white) | 0.004 | -0.373, 0.381 | 0.982 |
| **Infant characteristics:** |  |  |  |
| Temperament | 0.026 | -0.203, 0.254 | 0.819 |
| Intelligence | 0.000 | -0.005, 0.005 | 0.915 |
| **Parental SES:** |  |  |  |
| Parental education | -0.089* | -0.172, -0.005 | 0.039 |
| Family income-to-needs ratio | -0.038* | -0.073, -0.004 | 0.030 |
| **Child health status:** |  |  |  |
| Gestational age | -0.028 | -0.084, 0.028 | 0.311 |
| Breastfeeding history | 0.003 | -0.011, 0.016 | 0.693 |
| BMI percentile | 0.006*** | 0.003, 0.008 | <0.001 |
| General health rating | -0.041 | -0.208, 0.127 | 0.625 |
| **Early education indicator:** |  |  |  |
| Classroom emotional quality | -0.025 | -0.172, 0.123 | 0.732 |

*<.05, **<.01, ***<.001

SES=socioeconomic status; CMR=cardiometabolic risk; CI=confidence interval; PI=Pacific Islander; BMI=body mass index

Supplemental Table 4. Direct effects of the early educational indicator, classroom instructional quality, adjusted for socio-demographics, infant characteristics, parental SES, and child health status.

|  | **DV: CMR Composite** | | |
| --- | --- | --- | --- |
|  | **Beta** | **95% CI** | **p-value** |
| **PREDICTORS:** |  |  |  |
| **Socio-demographics:** |  |  |  |
| Biological sex (female vs. male) | -0.511*** | -0.673, -0.350 | <0.001 |
| Black (vs. white) | -0.008 | -0.344, 0.328 | 0.963 |
| Latino (vs. white) | 0.001 | -0.359, 0.361 | 0.997 |
| Asian/PI (vs. white) | -0.036 | -0.384, 0.311 | 0.835 |
| ‘Other’ (vs. white) | -0.002 | -0.389, 0.386 | 0.993 |
| **Infant characteristics:** |  |  |  |
| Temperament | 0.025 | -0.198, 0.249 | 0.815 |
| Intelligence | 0.000 | -0.004, 0.005 | 0.887 |
| **Parental SES:** |  |  |  |
| Parental education | -0.088* | -0.172, -0.004 | 0.040 |
| Family income-to-needs ratio | -0.038* | -0.073, -0.004 | 0.031 |
| **Child health status:** |  |  |  |
| Gestational age | -0.028 | -0.085, 0.028 | 0.314 |
| Breastfeeding history | 0.002 | -0.011, 0.016 | 0.705 |
| BMI percentile | 0.006*** | 0.003, 0.008 | <0.001 |
| General health rating | -0.042 | -0.210, 0.126 | 0.613 |
| **Early education indicator:** |  |  |  |
| Classroom instructional quality | -0.039 | -0.223, 0.145 | 0.656 |

*<.05, **<.01, ***<.001

SES=socioeconomic status; CMR=cardiometabolic risk; CI=confidence interval; PI=Pacific Islander; BMI=body mass index
